# Editorial: Bioresorbable materials: What is new?

**DOI:** 10.3389/fbioe.2026.1869987

**Published:** 2026-06-05

**Authors:** Nenad Filipović, Magdalena M. Stevanović, Shen Liu, Kai Zheng

**Affiliations:** 1 Institute of Technical Sciences of SASA, Group for Biomedical Engineering and Nanobiotechnology, Belgrade, Serbia; 2 Department of Orthopedic Surgery, Shanghai Sixth People’s Hospital Affiliated to Shanghai Jiao Tong University School of Medicine, Shanghai, China; 3 Jiangsu Province Engineering Research Center of Stomatological Translational Medicine, Nanjing Medical University, Nanjing, China

**Keywords:** bioresorbable implants, bioresorbable conductive composites, transient bioelectronics, magnesium alloys, bioresorbable polymers, coronary covered stents

## Introduction

Bioresorbable materials have emerged as a rapidly expanding class of biomaterials with the potential to significantly improve the safety and functionality of numerous medical devices. Unlike conventional permanent implants, bioresorbable materials are designed to perform their therapeutic or diagnostic roles and then degrade within the body into physiologically compatible products. This capability eliminates the need for secondary surgical procedures for implant removal, thereby reducing patient risk, healthcare costs, and long-term complications associated with permanent foreign materials. Despite significant progress in materials science, nanotechnology, and biomedical engineering, the design of bioresorbable materials remains a complex challenge. Materials must simultaneously satisfy several demanding criteria, including sufficient mechanical stability during the functional lifetime of the device, predictable and controllable degradation rates, and full biocompatibility of degradation products. Achieving these properties requires careful control of composition, microstructure, and surface characteristics, as well as a deep understanding of the interactions between biomaterials and biological environments.

The Research Topic “Bioresorbable Materials: What is New?” brings together contributions that collectively highlight recent advances in this dynamic research field. The included articles span both conceptual perspectives and experimental developments, covering biodegradable electronic materials, magnesium-based implants, and polymeric systems for cardiovascular applications.

## Overview of the research topic

Implantable electronic devices have become essential tools for physiological monitoring, therapeutic stimulation, and controlled drug delivery. One of the key challenges in their design is the development of bioresorbable electrodes that can provide reliable electrical performance while gradually degrading in physiological environments. To address this, fabrication strategies such as solvent casting, additive manufacturing, and electrospinning are continuously evolving, enabling the creation of flexible and conductive structures that can effectively interface with soft tissues. The excellent review article that highlights some of the recent progress in this field is given by Đorđević et al. The article covers the selection of conductive materials (including Mg, Zn, Mo, W, and polymer–metal composites), fabrication techniques, and a wide range of applications, such as electrophysiological monitoring, electrostimulation therapies, chemical sensing, and multifunctional theranostic systems. As such, it serves both as an excellent starting point for new researchers and as a concise resource for those already working in the field.

Similarly, the review by Chaudhari et al. summarizes recent advances in magnesium-based biomaterials, with a focus on nanoscale systems and surface engineering strategies to control corrosion and improve biological response. The authors discuss key approaches, including ceramic, polymer, and hybrid coatings, as well as alloying with elements such as Zn, Ca, Sr, and rare earths to tune mechanical properties and degradation behavior. It also addresses how material properties affect biological performance, particularly osteointegration and bone regeneration. By comparing published results with clinical needs and existing solutions, the review highlights current limitations and outlines future research directions, making it a useful guide for the design of next-generation orthopedic biomaterials.

The potential of Mg-based materials was further demonstrated in the original research article provided by Gmitro et al. In this study, the authors demonstrate how reinforcing magnesium with diopside-based bioactive glass nanoparticles and alloying it with Sc and Sr can improve corrosion resistance and biological performance. Importantly, the *in vivo* results support the material’s clinical potential. After 3 months of implantation in a rat femoral defect model, supported by *ex vivo* imaging, the material showed low corrosion, strong osteointegration, and clear new bone formation. Compared to the FDA-approved alloy WE43, the proposed material achieved similar or slightly better performance.

The final contribution in this Research Topic represents a proof-of-concept investigation of polymer-based membranes for cardiovascular applications. To coat coronary bare metal stents, the Rezvova et al. utilize electrospinning and bioresorbable polymers, including polycaprolactone (PCL), polydioxanone (PDO), poly (lactide-co-caprolactone) (PLCL), and poly (lactide-co-glycolide) (PLGA). A thorough characterization, combining physicochemical, mechanical, and biological evaluation (including stability under cyclic expansion and contraction, as well as hemocompatibility tests), confirms the robustness and functionality of the developed system. Low thrombogenic potential, demonstrated through platelet adhesion studies, together with favorable *in vivo* responses in the rat abdominal aorta (vascular wall regeneration after 20 days), further highlight the material’s potential. Among the tested systems, PLCL/PDO membranes showed particularly strong performance. Overall, these results provide a solid foundation for future preclinical studies of stent systems in larger animal models.

Taken together, the contributions included in this Research Topic illustrate the key trends and emerging directions in bioresorbable materials. From transient electronic systems to biodegradable metallic implants, particularly those based on Mg and Mo, including their alloying with rare elements and integration with polymers, and electrospun membranes for cardiovascular applications, these studies highlight both the central challenges and the significant potential of the field, especially in bridging the gap between material design and clinical application.

## Conclusions and outlook

Importantly, this collection of articles underscores the need for more comprehensive *in vitro* testing under realistic, dynamic conditions, enabling more reliable prediction of *in vivo* performance and helping to reduce the need for extensive animal studies. In this context, controlling degradation behavior, ensuring long-term functionality, and improving testing strategies remain critical steps toward clinical translation. Future progress in the field will depend not only on material innovation, but also on the establishment of robust regulatory and validation frameworks for increasingly complex bioresorbable systems. At present, bioresorbable biomaterials and medical devices are largely assessed using combinations of existing testing protocols for conventional material classes, focusing on biocompatibility, degradation behavior, mechanical stability, and long-term safety. However, the rapid emergence of multifunctional bioresorbable implants and transient systems continues to challenge current regulatory frameworks, which still lack dedicated classification and validation pathways capable of fully addressing the dynamic nature of these technologies. In parallel, greater attention should also be directed toward reproducible and scalable manufacturing strategies and quality control protocols capable of supporting reliable clinical translation of next-generation bioresorbable technologies.

